# Complete DNA Sequence of *Pseudomonas syringae* pv. actinidiae, the Causal Agent of Kiwifruit Canker Disease

**DOI:** 10.1128/genomeA.01054-15

**Published:** 2015-09-17

**Authors:** Matthew D. Templeton, Benjamin A. Warren, Mark T. Andersen, Erik H. A. Rikkerink, Peter C. Fineran

**Affiliations:** a The New Zealand Institute for Plant & Food Research Limited, Auckland, New Zealand; b School of Biological Sciences, University of Auckland, Auckland, New Zealand; c Department of Microbiology, University of Otago, Dunedin, New Zealand; d Bio-Protection Research Centre, Lincoln University, Canterbury, New Zealand

## Abstract

*Pseudomonas syringae* pv. actinidiae is the causal agent of bacterial canker of kiwifruit, a disease that has rapidly spread worldwide. We have fully sequenced and assembled the chromosomal and plasmid DNA from *P. syringae* pv. actinidiae ICMP 18884 using the PacBio RS II platform.

## GENOME ANNOUNCEMENT

*Pseudomonas syringae* pv. actinidiae is the causal agent of bacterial canker of kiwifruit, a disease that has particularly devastated plantings of *Actinidia chinensis* throughout the world (1–3). The speed and severity of the *P. syringae* pv. actinidiae pandemic has led this pathogen to become a model for the rapid emergence of new diseases (4). Many draft *P. syringae* pv. actinidiae genomes from a range of geographical locations have been sequenced and deposited in GenBank (5–8). We have fully sequenced and assembled the genome of *P. syringae* pv. actinidiae ICMP 18884 using the single-molecule real-time (SMRT) PacBio RS II platform (http://www.pacificbiosciences.com/). The read coverage averaged between 150- and 250-fold. These reads were assembled using the Hierarchical Genome Assembly Process algorithm into a chromosome of 6,580,291 bp and a plasmid of 74,423 bp (http://www.pacificbiosciences.com/). The *P. syringae* pv. actinidiae ICMP 18884 assembled sequence and individual reads were submitted under BioProject no. PRJNA71845 and BioSample SAMN02727983. The genome was annotated using the NCBI Prokaryotic Genome Annotation Pipeline (PGAP) (http://www.ncbi.nlm.nih.gov/genome/annotation_prok), and 5,982 genes, which included 5,761 coding sequences, 140 pseudogenes, and 81 RNA-encoding genes, were identified. The genome was also annotated using the RAST webserver (http://rast.nmpdr.org/) (9), which predicted 6,126 coding sequences. The two methods generally gave identical gene calls; however, a class of genes that were inconsistently predicted by both programs were the type III secreted effectors (T3SE). Since T3SEs are some of the most important genes for understanding how *P. syringae* pv. actinidiae causes disease, these were manually annotated based on gene sequences curated at the *P. syringae* Genome Resources site (http://www.pseudomonas-syringae.org/), and the corrected PGAP annotation was deposited in GenBank.

The high read coverage enabled methylation modifications to be annotated, and 16,639 4-methyl-cytosine (4 mC) and 4,835 6-methyl-adenosine (6 mA) residues were identified. This is the first time that methylated residues have been reported for a genome from *P. syringae*. Analysis of the annotated genome predicted the presence of seven restriction modification systems. These were matched to two of the methylation motifs generated from the SMRT data using the REbase Web server (10). The results predict that the type I restriction enzyme system encoded by genes IYO_000025-35 cleaves at the 7-base AGCANNNNNGTC motif (underlined nucleotides indicate 6 mA sites), and the type II restriction system encoded by IYO_000855 recognizes the six-base palindrome CTCGAG (underlined nucleotides indicate 4 mC sites). AGCANNNNNGTC is a new restriction enzyme recognition site, and 99% of the 1,176 motifs present in the *P. syringae* pv. actinidiae genome were methylated at both target nucleotides. In contrast, only 54% of the target nucleotides in the CTCGAG motif are methylated. Interestingly, only about 10% of the methylated residues in the genome can be accounted for by these two motifs. This suggests either that there are other unrecognized restriction/methylation systems active in *P. syringae* pv. actinidiae not detected by REbase, or that the methylated residues play other roles in the genome, such as gene regulation. In some animal pathogens, adenosine methylation is required for the expression of some virulence genes (11).

### Nucleotide sequence accession numbers.

These sequences have been deposited in GenBank under the accession numbers CP011972 (chromosome) and CP011973 (plasmid). The versions described in this paper are the first versions.
